# Effect of Graphene Oxide Nanoparticles Incorporation on the Mechanical Properties of a Resin-Modified Glass Ionomer Cement

**DOI:** 10.3390/polym16172401

**Published:** 2024-08-24

**Authors:** Rafael Ubaldo Moreira e Moraes, Marcos Andre Pinheiro Abreu, Mayara Cristina Abas Frazão, Paulo Vitor Campos Ferreira, José Bauer, Ceci Nunes Carvalho, Edilausson Moreno Carvalho

**Affiliations:** 1 Dentistry Postgraduate Program, University Ceuma, R. Josué Montello, 1, Renascença II, São Luís 65075-120, Brazil; 2 School of Dentistry, University Ceuma, R. Josué Montello, 1, Renascença II, São Luís 65075-120, Brazil; 3 Dentistry Biomaterials Laboratory (Biomma), School of Dentistry, Federal University of Maranhão (UFMA), São Luís 65080-805, Brazil

**Keywords:** graphene oxide, glass ionomer cement, mechanical tests

## Abstract

The objective of this study was to evaluate the effect of incorporating different concentrations of graphene oxide (GO) nanoparticles on the mechanical properties of a resin-modified glass ionomer cement (RMGIC). A commercial RMGIC (Resiglass R, Biodinâmica) was modified by incorporating 0.1% and 0.5% (by weight) of GO into the powder’s material. An unmodified RMGIC was used as a control group. Powder samples were characterized using Scanning Electron Microscopy (SEM) and Energy Dispersive Spectroscopy (EDS). Specimens were fabricated and subjected to flexural strength (*n* = 15), modulus of elasticity (*n* = 15), Vicker’s microhardness (*n* = 10), and surface roughness tests (*n* = 10). Data were analyzed using one-way ANOVA and Tukey’s post hoc test (α = 5%). Experimental groups’ powder demonstrated a homogeneous dispersion of GO. No statistically significant difference was observed in flexural strength (*p* = 0.067) and modulus of elasticity (*p* = 0.143) tests. The groups containing 0.1% and 0.5% GO showed significantly higher microhardness and lower surface roughness values (*p* < 0.001) compared to the control group. The incorporation of GO nanoparticles at concentrations of 0.1% and 0.5% improved the microhardness and surface roughness without negatively affecting the flexural strength and modulus of elasticity of an RMGIC.

## 1. Introduction

Glass ionomer cements (GICs) have been widely used in dentistry across various specialties since their development in the 1970s [1]. This material is considered a first choice in conservative treatments due to its good biocompatibility with oral tissues and fluoride release [2]. Currently, GICs are available in different formulations, with conventional GICs and resin-modified GICs (RMGICs) being prominent [3].

Conventional GICs result from an acid-base setting reaction, consisting of the reaction product between weak polymeric acids and basic glass powders. These materials exhibit desirable properties such as adhesion to dental tissues, a linear thermal expansion coefficient similar to that of dentin, and fluoride release in the oral cavity [4]. However, this type of cement has some limitations, such as low mechanical properties, susceptibility to wear and fractures, and technical sensitivity during handling and insertion [3,4,5].

To improve the physicochemical and mechanical properties of GICs, various modifications to the basic composition of these materials have been proposed, notably the development of resin-modified GICs (RMGICs) [6]. In this category of GICs, the primary modification was the addition of polymerizable organic monomers, commonly hydroxyethyl methacrylate (HEMA), providing an additional (dual) setting reaction that can be self-activated and/or light-activated [6,7].

The literature has shown that RMGICs have better mechanical and aesthetic properties, in addition to easy manipulation, when compared to conventional GICs [8]. Malhotra et al. [9] reported superior compressive and flexural strength results of RMGICs to those found in conventional GICs. Clinical studies also indicated better clinical performance of these materials. Chadwik and Evans [10] evaluated the failure rates of class II restorations in primary teeth, and significantly lower rates were found for RMGICs. Dülgergil et al. [11] reported better clinical performance of RMGICs in Atraumatic Restorative Treatment (ART), when compared to conventional GICs.

Although RMGICs exhibit better properties compared to conventional GICs [12], evidence has shown that their performance, especially clinically, may be inferior to that observed with the use of other resin-based restorative materials, such as composite resins [13]. Thus, studies have proposed the addition of various particles to RMGICs, acting as reinforcing agents to improve the properties of these materials [14,15].

Graphene is a carbon allotrope consisting of a layer of atoms arranged in a honeycomb pattern, with high mechanical strength and modulus of elasticity [16]. Graphene and its derivatives, such as graphene oxide (GO), exhibit good biocompatibility compared to other forms of carbon, allowing their inclusion in biomaterials to increase strength and improve the mechanical properties of composites and nanocomposites [17]. Furthermore, graphene-based materials have significant antimicrobial potential, acting against Gram-positive and Gram-negative bacteria [18,19]. This effect results from graphene’s ability to physically damage microorganisms by penetrating and cutting the cell membrane, causing irreversible damage [17].

Given these properties, the incorporation of graphene nanoparticles has been proposed as an alternative for the development of biocompatible materials with low cytotoxicity and capable of stimulating cell differentiation [20]. Several studies have shown that GO can be successfully added to biomaterials with different applications, such as the production of scaffolds for bone and pulp tissue regeneration [17,21], periodontal tissue regeneration [22], implant coatings [23,24], cements [25,26] and resin-based materials [27].

Although the addition of graphene is a promising strategy for developing dental restorative materials with enhanced properties, there is little evidence in the literature regarding the inclusion of these particles and their derivatives in RMGICs. Few studies have shown that the addition of graphene can improve some properties of RMGICs, such as flexural strength [28,29] and shear strength in dentin [30]. However, factors such as the composition and concentration of these nanoparticles, in addition to the association with other fillers, still raise doubts about the real impact of the addition of GO in RMGICs.

Thus, this study aimed to evaluate the effect of incorporating different concentrations of graphene oxide nanoparticles on the mechanical properties of an RMGIC. The null hypotheses tested were that the addition of GO nanoparticles does not influence the (I) compressive strength, (II) modulus of elasticity, (III) microhardness, and (IV) surface roughness of the RMGIC.

## 2. Materials and Methods

### 2.1. Preparation of Resin-Modified Glass Ionomer Cements with Graphene Oxide

For this study, the resin-modified glass ionomer cement (RMGIC) Resiglass R (Biodinâmica, Ibiporã, Paraná, Brazil) was used. Graphene oxide (GO, Sigma Aldrich, St. Louis, MI, USA) particles were incorporated at two different concentrations (0.1% and 0.5%) into the RMGIC powder. The liquid was used without modifications and in the same proportions recommended by the manufacturer. The cements were then divided into three experimental groups: Group I: control—Resiglass R (without modifications), Group II: Resiglass R + 0.1% GO, and Group III: Resiglass R + 0.5% GO.

**Figure 1:**
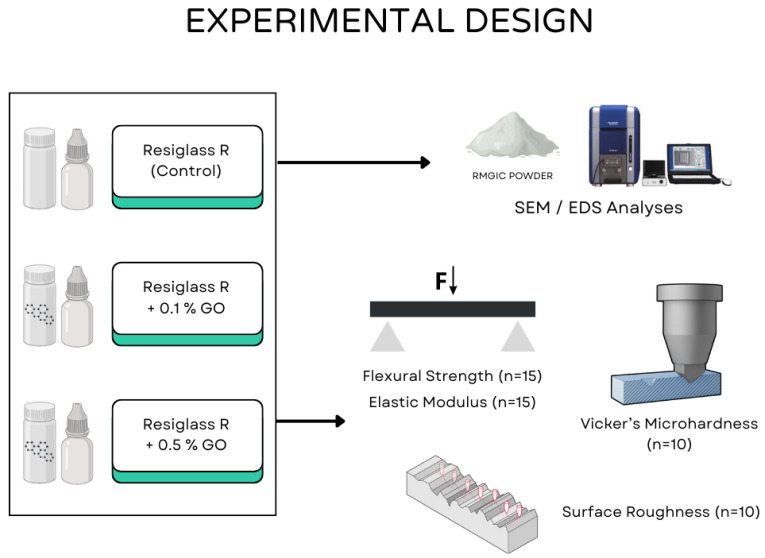
Study’s experimental design.

After the preparation of the experimental cements, powder samples from the experimental groups were characterized using Scanning Electron Microscopy (SEM) and Energy Dispersive Spectroscopy (EDS). SEM images (TM3030, Hitachi, Tokyo, Japan) at 1000× magnification were obtained for morphological analysis of the particles. Subsequently, EDS spectra (Quantax, Bruker, Billerica, MA, USA) were collected in a manner similar to the SEM images for the identification of the elemental profile of the materials. The study’s experimental design is summarized in Figure 1.

### 2.2. Flexural Strength Test

To fabricate the specimens (*n* = 15), a split stainless-steel mold with the following internal dimensions was used: 10 mm in length, 2 mm in width, and 2 mm in height. During the fabrication of the specimens, the mold was placed on a polyester strip and a 1 mm thick glass slide and filled with the materials according to the study’s experimental groups. Then, a second polyester strip and glass slide were placed with light pressure on the mold to standardize the filling of the mold with the material. The samples were then light-cured for 40 s on each side (VALO, Ultradent, Indaiatuba, São Paulo, Brazil). Any excess material was removed with a scalpel. The specimens were then stored in distilled water in hermetically sealed containers for 24 h at 37 °C in an oven (ISO Standard 9917-2:2017 [31]).

Before testing, the specimens were measured with a digital caliper (ABSOLUTE Digimatic, Mitutoyo Corporation, Kawasaki, Kanagawa, Japan). The three-point bending test was performed on a universal testing machine (3342, Instron, Norwood, MA, USA). The distance between supports was 6 mm, and the test speed was 1 mm/min. The flexural strength was calculated using the following formula:

$$\mathrm{FS}=3\mathrm{Fl}\frac{3\mathrm{Fl}}{2{\mathrm{bh}}^{2}}$$

where FS is the flexural strength (MPa), F is the load required for fracture, l is the distance between the supports, and b and h are the width and height of the specimen (mm), respectively.

### 2.3. Elastic Modulus Test

The data used to obtain the elastic modulus were taken from the flexural strength test. During the test, a computer connected to the testing machine recorded four load values corresponding to the displacements of the active tip (0.01 mm, 0.03 mm, 0.05 mm, and 0.07 mm) for each specimen. Each load value and the corresponding displacement were inserted into the formula described below, obtaining four modulus of elasticity (ME) values for each sample, which were then averaged (ISO Standard 4049:2019 [32]):

$$\mathrm{ME}\;=\frac{{\mathrm{Fl}}^{3}}{4{\mathrm{bh}}^{3}d}$$

where F is the load recorded at the moment, l is the distance between the supports, b and h are the width and height of the specimen (mm), respectively, and d is the deflection (mm) corresponding to F. The values obtained were expressed in GPa.

### 2.4. Vickers Microhardness Test (VHN)

Ten disc-shaped samples (5 mm in diameter ×2 mm in height) were fabricated for each group (*n* = 10). The specimens were then embedded in PVC tubes with clear self-curing acrylic resin (Clássico Produtos Odontógicos, São Paulo, São Paulo, Brazil), leaving only the outer surface exposed. To ensure the maintenance of parallelism during cutting, the samples were fixed on a glass plate using double-sided tape. The PVC tube was placed around the samples, the resin powder was added to cover the samples, and the monomer was dripped until the powder was saturated.

After this period, the embedded specimens were identified and subjected to finishing and polishing on a polishing machine (AROTEC, Cotia, São Paulo, Brazil) using decreasing abrasion order of sandpapers: 400, 600, 1000, 1200, and 2000, under cooling. The specimens were then taken to a microhardness tester (HMV-2, Shimadzu, Tokyo, Japan) equipped with a Vickers indenter. Measurements were made on the exposed surface at three random points in the center of the sample, with a load of 100 g for 10 s. The average values obtained for each specimen were recorded and used for statistical analysis.

### 2.5. Surface Roughness Test

Ten disc-shaped samples (10 mm in diameter ×1 mm in height) were fabricated for each group (*n* = 10) in the same manner described in Section 2.4. The surface roughness of the specimens was measured with a profilometer (precision of 300 mm, speed of 0.5 mm/s, and five cut-off points of 0.8 mm—SJ301, Mitutoyo Corporation, Kawasaki, Kanagawa, Japan). Three readings were taken on each sample, and an average Ra value (arithmetic mean deviation of the roughness profile) was calculated in microns. After the test was performed, the surface of the samples was analyzed using a SEM (TM3030, Hitachi, Chiyoda, Tokyo, Japan) at 500× magnification.

### 2.6. Statistical Analysis

Statistical analysis was performed using SigmaPlot software (SigmaPlot 12.0, Systat Software Inc., San Jose, CA, USA). The data were subjected to the Shapiro-Wilk normality test (α = 0.05). Flexural strength, modulus of elasticity, Vickers microhardness, and surface roughness data were subjected to one-way ANOVA with a Holm-Sidak post hoc test, adopting a significance level of 5%. The results of the powder characterization analyses by SEM/EDS were evaluated qualitatively.

## 3. Results

### 3.1. Powder Characterization (SEM/EDS)

**Figure 2:**
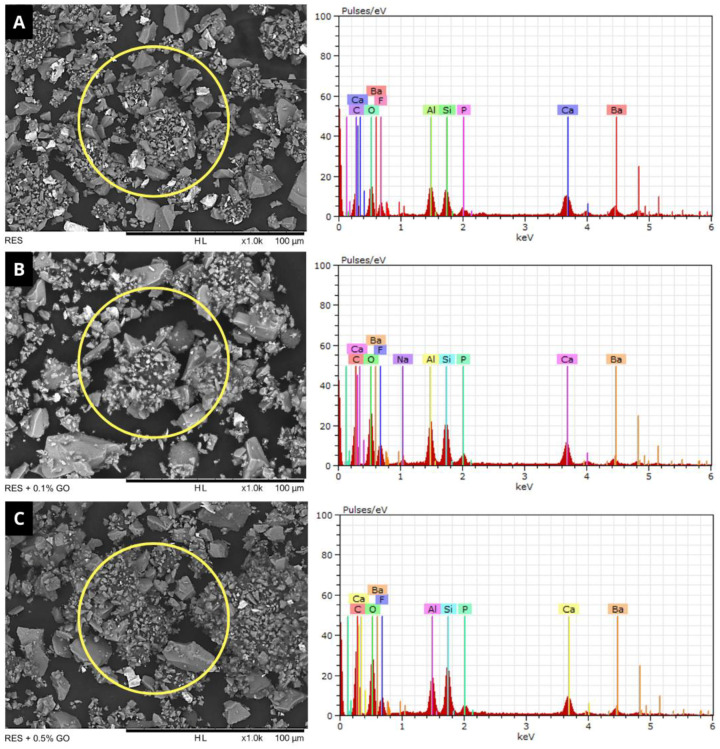
SEM images and EDS spectra of powder samples from the evaluated experimental groups. (**A**): Resiglass R; (**B**): Resiglass R + 0.1% GO; (**C**): Resiglass R + 0.5% GO. (Magnification: ×1000; Scale Bar: 100 µm).

SEM images and EDS spectra of powder samples from the experimental groups are presented in Figure 2. SEM analysis showed that the groups containing GO nanoparticles exhibited a visual appearance similar to that observed in the control group (Figure 2A), confirming a homogeneous dispersion of graphene into the RMGIC powder.

The EDS spectra demonstrated an increase in the intensity of carbon (C) and oxygen (O) peaks in the groups containing 0.1% (Figure 2B) and 0.5% (Figure 2C) GO, confirming the presence of these nanoparticles in the material powder. In addition to these elements, samples also presented fluorine (F), calcium (Ca), silicon (Si), aluminum (Al), barium (Ba), phosphorus (P), and sodium (Na). The quantitative analysis in the EDS spectra is shown in Table 1.

### 3.2. Flexural Strength and Elastic Modulus

**Figure 3:**
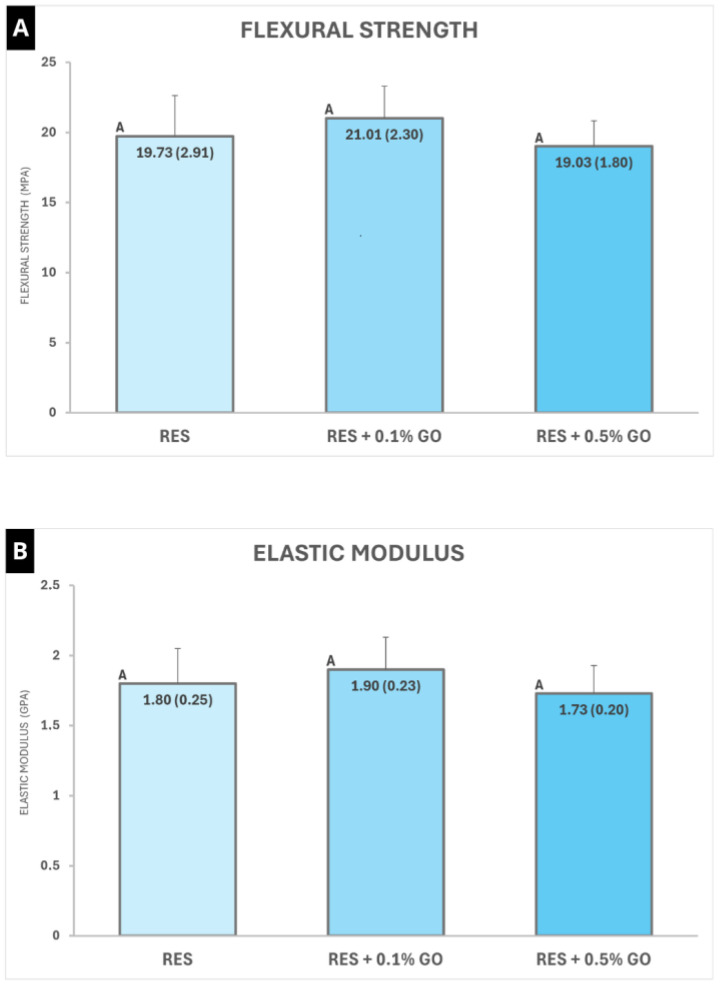
Mean values of flexural strength (**A**) and modulus of elasticity (**B**) of the materials evaluated. Equal capital letters indicate that there is no statistical difference between the groups (*p* > 0.05).

The results of the flexural strength and modulus of elasticity tests are presented in Figure 3. In both tests, no statistically significant difference was observed between the control and experimental groups (*p* = 0.067 and *p* = 0.143, respectively).

### 3.3. Vickers Microhardness

**Figure 4:**
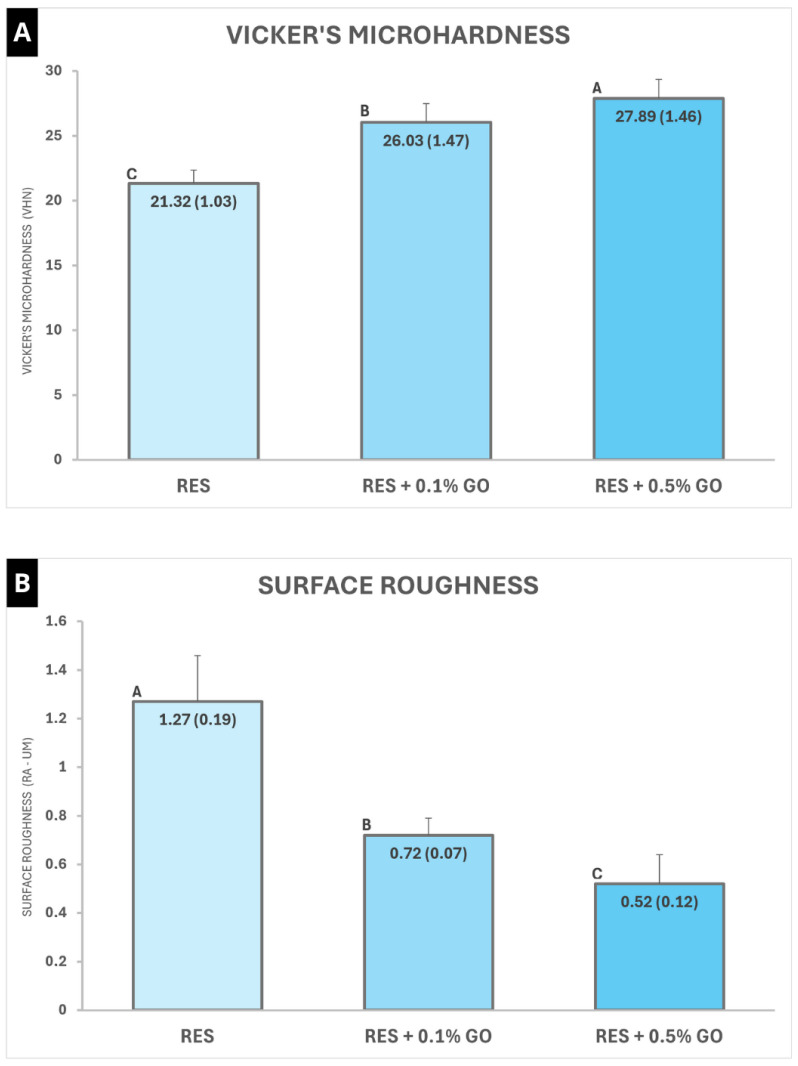
Mean and standard deviation values of Vicker’s microhardness (**A**) and surface roughness (**B**) results. Different capital letters indicate a statistically significant difference (*p* < 0.05).

The microhardness analysis results are presented in Figure 4A. The incorporation of GO nanoparticles at concentrations of 0.1% and 0.5% significantly increased the VHN values (*p* < 0.001) compared to the control group. The group containing 0.5% GO showed the highest values among the evaluated groups.

### 3.4. Surface Roughness

The incorporation of GO nanoparticles at both concentrations significantly reduced the surface roughness values of the RMGIC (*p* < 0.001) compared to the control group (Figure 4B). The group containing 0.5% GO presented the lowest roughness values among the evaluated groups.

**Figure 5:**
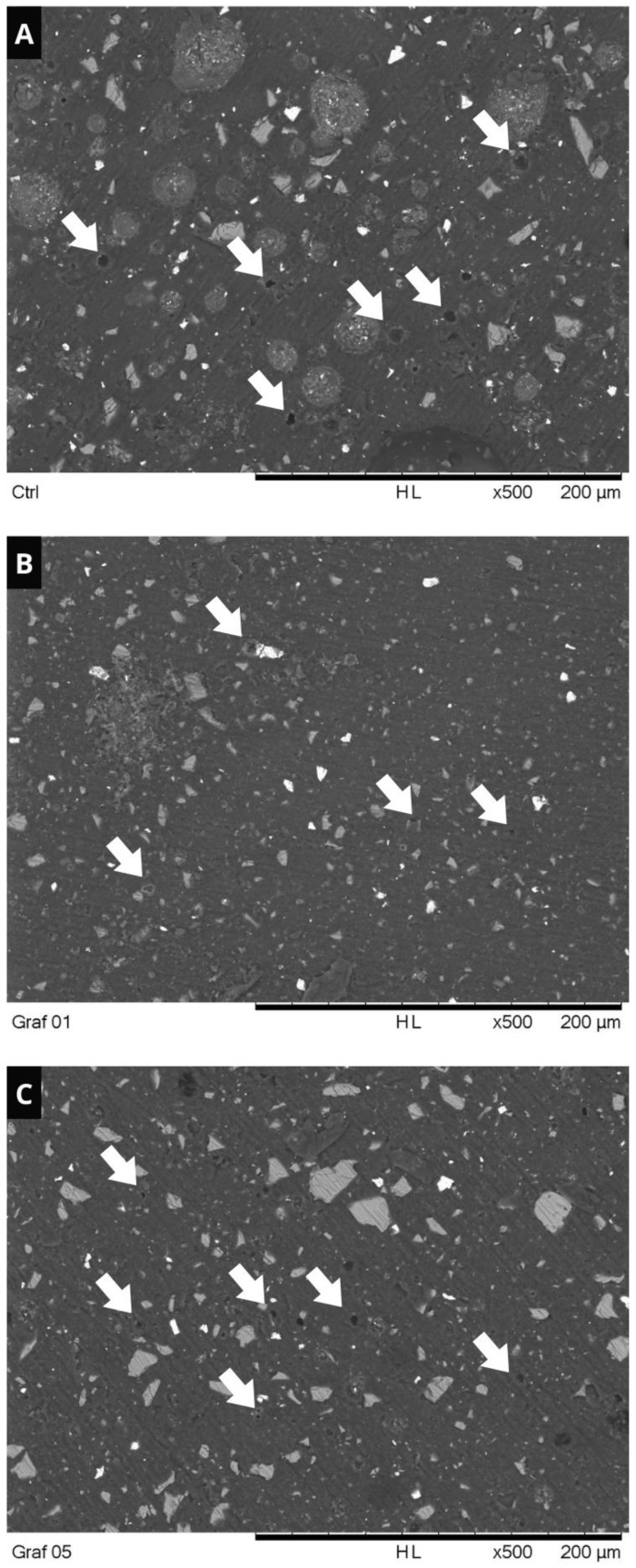
Representative SEM images of the surface roughness test samples. (**A**): control. (**B**): 0.1% GO. (**C**): 0.5% GO. White arrows: surface porosities. (Magnification: ×500; Scale Bar: 200 µm).

Representative SEM images of the samples subjected to the surface roughness test are shown in Figure 5. It was possible to observe that the samples from the control group (Figure 5A) presented larger and deeper porosities (white arrows), unlike the groups containing 0.1% (Figure 5B) and 0.5% GO (Figure 5C).

## 4. Discussion

The results of this study demonstrated that the addition of graphene oxide (GO) nanoparticles to the powder of the material did not promote improvements in flexural strength and modulus of elasticity, accepting the first and second null hypotheses. On the other hand, a significant increase in microhardness and a decrease in surface roughness were observed in the groups containing 0.1% and 0.5% GO, thereby rejecting the third and fourth null hypotheses.

Graphene oxide is commonly obtained from graphite and synthesized through chemical processes with reasonable cost-effectiveness [33,34]. However, the presence of functional groups, such as ketonic, carboxylic, epoxy, and hydroxyl groups, can alter the chemical compatibility [35] of GO, complicating the incorporation of these nanoparticles into other materials.

In this study, SEM and EDS analyses were performed to evaluate the dispersion pattern of the nanoparticles in powder samples from the experimental groups. The results demonstrated that at both concentrations, no clusters of GO were observed in the RMGIC powder, presenting a visual appearance similar to that of the control group (Figure 2A). Additionally, there was an increase in the intensity of the C peak in the groups containing RMGIC+GO. The addition of 0.1% and 0.5% GO to the RMGIC powder promoted an increase of approximately 4% and 13%, respectively, when compared to the control (Table 1). These findings are consistent with results from other studies that evaluated the incorporation of graphene derivatives into dental materials at concentrations up to 4% [26,36,37], demonstrating the compatibility of these nanoparticles with RMGIC powder.

The addition of particles to the composition of GICs to improve the mechanical properties of these materials has been reported in the literature for many years [38]. The first modifications to the basic composition of GICs were made by incorporating microparticulate powders of various metal alloys, such as silver, aluminum, chromium, and nickel [39]. Although some studies have demonstrated a positive effect of metal incorporation into conventional GICs [40,41], the literature generally indicates that this reinforcement does not significantly improve important mechanical properties such as compressive and flexural strength [42].

More recently, the effect of nanoparticle addition on the properties of GICs has been studied. Several studies have proposed the incorporation of particles such as titanium dioxide (TiO_2_) [43], alumina [44], zirconia [45], and hydroxyapatite [46] with varying degrees of success. Elsaka et al. [43] added 3% and 5% TiO_2_ concentrations to a GIC and observed a significant increase in flexural strength when compared to the control. The authors highlight that the observed results can be attributed to the small size of the TiO_2_ particles mixed with the glass powder of the material, increasing the range of particle size distribution and acting as additional bonding sites for the polyacrylic polymer, thereby reinforcing the GIC. Similarly, Khademolhosseini et al. [44] and Alatawi et al. [46] highlight that the cross-linking formation during setting plays an important role on the mechanical properties of the cements. It is worth noting that in these studies, using low concentrations of nanoparticles, such as 1%, it was not possible to observe improvements in the mechanical properties, especially flexural strength.

Regarding mechanical properties, conflicting results have been observed in the literature, with some studies demonstrating a positive effect [44,45] or no significant improvement [47] in properties such as flexural strength and modulus of elasticity. In the present study, the incorporation of 0.1% and 0.5% GO did not alter the mean values of flexural strength and modulus of elasticity of the material. These results are consistent with other studies that evaluated the modification of commercial RMGICs with nanoparticles at lower concentrations [36,47,48]. Although some more recent studies have demonstrated that the addition of GO nanoparticles can improve the flexural strength of RMGICs, these studies used minimum concentrations of 1% [29,30]. Nicholson et al. [42] emphasize that factors such as the type of nanoparticle, concentration, and composition of the RMGIC can significantly influence the effect of adding these elements on the material’s mechanical properties.

On the other hand, the findings of this study demonstrated a significant improvement in the surface properties of the experimental groups, with the greatest positive effect observed in the RMGIC + 0.5% GO group. Nanoparticles, when used as reinforcement particles, can fill small spaces between microparticles, leading to a significant reduction in the material’s porosity [48]. This mechanism may have contributed to the observed increase in microhardness and decrease in surface roughness in the groups containing 0.1% and 0.5% GO. Although limited evidence is available in the literature, these findings are consistent with similar studies that evaluated the effect of incorporating graphene derivatives into GICs [26,36]. 

Previous studies that evaluated the effect of adding other particles, such as chitosan, to GICs observed similar results to those reported in this study [49,50]. The authors emphasize that the particle size distribution influences the roughness of these materials: the smaller the particle size is, the better polishability will be obtained in these materials, leading to lower Ra values. In addition, the presence of nanoparticles can lead to the formation of smaller porosities on the surface of the material [49]. These findings corroborate the results of the surface’s samples subjected to the roughness test, represented in Figure 5, where shallower and smaller porosities were observed in the groups containing 0.1% and 0.5% GO (Figure 5B,C), when compared to the control (Figure 5A).

Despite the incorporation of GO presenting a beneficial strategy for improving the surface properties of RMGICs, some limitations should be considered. These nanoparticles exhibit a characteristic dark color, which may cause significant color changes when added to other materials. In the present study, even the incorporation of GO at the lowest concentration (0.1%) resulted in a significant darkening of the RMGIC, which could negatively affect the polymerization process. Therefore, in preliminary tests, a concentration of 0.5% was defined as the limit for adding these particles. Additionally, the authors are not aware of other studies that explored the impact of GO on resin-modified glass ionomers. Thus, further laboratory studies should be conducted to evaluate other important properties, such as the biocompatibility of these modified cements.

## 5. Conclusions

The incorporation of GO nanoparticles at concentrations of 0.1% and 0.5% increased the microhardness and decreased the surface roughness without affecting the flexural strength and modulus of elasticity of an RMGIC. Incorporating GO nanoparticles may be a promising strategy for the development of RMGICs with improved surface properties.

## Figures and Tables

**Table 1:** Distribution (in percentage) of the elements found in the EDS analyses.

Elements	RES	RES + 0.1% GO	RES + 0.5% GO
Carbon	27.52%	31.91%	40.30%
Oxygen	26.81%	30.73%	31.22%
Fluorine	10.27%	11.99%	9.19%
Barium	8.65%	4.95%	3.00%
Aluminum	8.52%	5.58%	4.80%
Calcium	8.06%	6.63%	3.91%
Silicon	7.91%	6.07%	6.28%
Phosphorus	2.26%	1.40%	1.30%
Sodium	-	0.75%	-

## Data Availability

The original contributions presented in the study are included in the article, further inquiries can be directed to the corresponding author.
